# An Epidemiological Investigation and Drug-Resistant Strain Isolation of *Nematodirus oiratianus* in Sheep in Inner Mongolia, China

**DOI:** 10.3390/ani13010030

**Published:** 2022-12-21

**Authors:** Yang Liu, Penglong Wang, Rui Wang, Junyan Li, Bintao Zhai, Xiaoping Luo, Xiaoye Yang

**Affiliations:** 1 Key Lab of Ministry of Education for the Protection and Utilization of Special Biological Resources in Western China, Ningxia University, Yinchuan 750021, China; 2 School of Life Sciences, Ningxia University, Yinchuan 750021, China; 3 Department of Veterinary Parasitology, College of Veterinary Medicine, China Agricultural University, Beijing 100193, China; 4 College of Veterinary Medicine, Inner Mongolia Agricultural University, Hohhot 010018, China; 5 Inner Mongolia Academy of Agriculture and Animal Husbandry Sciences, Hohhot 010030, China; 6 Key Laboratory of Veterinary Pharmaceutical Development, Lanzhou Institute of Husbandry and Pharmaceutical Sciences, Chinese Academy of Agricultural Sciences, Ministry of Agriculture, Lanzhou 730050, China

**Keywords:** *Nematodirus oiratianus*, larval migration inhibition test, anthelmintic resistance, morphology, ITS-rDNA

## Abstract

**Simple Summary:**

*Nematodirus* has a serious impact on the gastrointestinal health of ruminants and the economic development of animal husbandry. This study investigated *Nematodirus* infections of different rearing patterns in sheep in two well-known natural pastures in Inner Mongolia, China. The results indicated that the average infection rate was more than 50%. Then, a naturally occurring strain of *Nematodirus* was obtained by using numerous isolation methods. We recorded the detailed development process of the nematode eggs and tested for drug resistance, and the results showed that the isolated strains were found to be ivermectin-resistant strains. Our study provides useful data for the morphological study of *Nematodirus* and the prevention and control of nematodes, offering valuable species resources for studying anthelmintic resistance in nematodes.

**Abstract:**

*Nematodirus* causes major economic losses in the development of the livestock industry, as they are common gastrointestinal parasites of cattle, sheep, and other ruminants. The present study investigated *Nematodirus* infections in sheep in the Hulunbuir and Xilingol Grasslands, two well-known natural pastures in Inner Mongolia, China. The results indicated that the average infection rate was more than 50%. Furthermore, a naturally occurring strain of *Nematodirus* was obtained using numerous isolation methods in the survey region. Conventional morphology and molecular biology were used to identify the strain. The larval migration inhibition test was used to determine the ivermectin level in the isolated strain. The results indicated that the larvae belonged to the species *Nematodirus oiratianus*. The strain was found to be ivermectin-resistant. Thus, these results recorded the detailed development processes of *Nematodirus* eggs, providing novel insights regarding the prevention and control of nematodes by using conventional anthelmintic regimens and by offering valuable species resources for studying anthelmintic resistance in nematodes.

## 1. Introduction

The livestock industry is relatively developed in Inner Mongolia, China. The Hulunbuir and Xilingol Grasslands are two well-known natural pastures that contain both a vast number of sheep and complex and diverse parasitic diseases. In particular, sheep gastrointestinal nematodes have a high infection rate and intensity of infection, and have caused substantial economic losses to the livestock industry. Among them, *Nematodirus* and *Haemonchus contortus* are the most serious parasites.

*Nematodirus* are soil-borne parasites that do not require an intermediate host for their development. These parasites primarily obtain nutrients by consuming the blood of their host and are common gastrointestinal nematodes that are a serious threat to livestock. *Nematodirus* is distributed worldwide. Oliver et al. [1] investigated *N. filicollis* and *N. spathiger* in 50 sheep farms in New Zealand and found infection rates of 76% and 100%, respectively. Hyuga and Matsumoto [2] found that the *Nematodirus* infection rate among alpaca in captivity in Japan was 13.2%. Jelinski et al. [3] investigated that the *Nematodirus* infection rate among calves in central Canada was 1.7%. Most areas in China have reported nematodiriasis, especially in Inner Mongolia, where the infection rate among sheep is 65.6% [4]. The infected animals develop anemia and diarrhea and can even die. Current treatment of this parasitic disease relies heavily on the use of ivermectin and other anthelmintics. However, abuse of such commonly used anthelmintics has led to the emergence of anthelmintic resistance in *Nematodirus* [5,6], which is an important factor limiting disease prevention and control.

At present, reports on *Nematodirus* and their resistance to anthelmintics are insufficient. An epidemiological study of *Nematodirus* in sheep on several pastures in the Hulunbuir and Xilingol Grasslands of Inner Mongolia, China, was conducted to understand the current condition of *Nematodirus* infection in sheep. Furthermore, anthelmintic treatment was administered in spring and autumn to identify and isolate anthelmintic-resistant strains. Using a combination of morphology, molecular biology, and larval migration inhibition test (LMIT), a resistant strain of *N. oiratianus* was successfully isolated and identified.

## 2. Materials and Methods

### 2.1. Ethics Approval

The study design was reviewed and approved by the Animal Ethics Committee of Ningxia University (permit no. 22-031). The procedures involving animals were carried out in accordance with the Animal Ethics Procedures and Guidelines of the People’s Republic of China. All efforts were made to minimize suffering and to reduce the number of sheep used in the experiment.

### 2.2. Epidemiological Investigation of Nematodiriasis in Sheep in Major Pastures in Inner Mongolia, China

The Hulunbuir and Xilingol Grasslands were used as the survey regions, and three pastures were selected from each region (A, B, C and D, E, F). Among them, the Hulunbuir New Barag Right Banner pasture flock was raised by semi-grazing and semi-feeding while the Xilinhot Blue Banner pasture flock was completely raised by grazing. Samples were collected at two instances: before anthelmintic treatments in spring and in autumn. Sixty sheep were selected from each pasture. Fresh feces were collected from sheep rectum, the sheep were marked with numbers, and the times and locations were recorded.

A small amount of saturated saline was added to 2 g of feces to be tested and ground. After 30 min, saturated saline was added until 58 mL, mixed well, and filtered through a 60-mesh sieve. The filtrate was poured into an ampoule, without overflowing, until a convex liquid surface formed. After 30 min, the convex liquid surface was dipped by a coverslip in the ampoule and then placed on a glass slide [7]. *Nematodirus* eggs were examined using a microscope (CX22, OLYMPUS, Tokyo, Japan).

*Nematodirus* infection rates for each pasture were calculated twice. SPSS 20.0 software was used to perform one-way analysis of variance and significance analysis.

### 2.3. Isolation and Identification of Naturally Occurring Nematodirus

#### 2.3.1. Morphological Identification

Based on the results of previous epidemiological studies, large amounts of fresh feces were collected from sheep infected with *Nematodirus*. Saturated saline was added to 50 g of fresh feces, and the mixture was ground and filtered through a 60-mesh sieve into a petri dish such that the filtrate protruded from the wall of the dish without overflowing. The filtrate was collected by touching a plastic coverslip to the convex liquid surface (without bubbles). After 20 min, the *Nematodirus* eggs on the coverslip were washed into a beaker containing saturated saline. This solution was then consecutively filtered through 100-mesh and 200-mesh sieves, leaving the eggs on the 200-mesh sieve. The eggs were then washed with distilled water, stored in a cell culture flask, and placed in an incubator at a constant temperature of 27 °C for 2 weeks. The development of the eggs was observed every day, and the transverse and longitudinal diameters were measured until they reached L3 [8].

The experimental sheep were newborn lambs from our laboratory. The animal was housed in a single pen and had free access to food and water. Fecal samples were collected and examined using the McMaster technique at regular intervals to ensure that the nematode egg counts of the sheep showed negative values (mean fecal egg count = 0 eggs per gram). A lamb without any nematode infections was selected, and 10,000 infectious *Nematodirus* L3 were administered once through the mouth. On the 25th day after artificial infection, *Nematodirus* eggs were found in the feces of the lamb [9]. The lamb was then dissected, and the wall and contents of the small intestine were washed and examined. Adult *Nematodirus* worms were collected and washed with normal saline. Some of these were collected and placed on a glass slide. Ten microliters of Lugol’s iodine solution was added [10], and the morphological characteristics of the parasites were observed using a microscope (CX22, OLYMPUS, Japan). The other adult worms were stored at −20 °C for use in molecular biology experiments.

#### 2.3.2. DNA Extraction, PCR, and Sequencing

Genomic DNA of adult *Nematodirus* was extracted using a commercial DNeasy Blood and Tissue kit (Qiagen, Hilden, Germany) according to the manufacturer’s instructions and stored at −20 °C until use. Internal transcribed spacer (ITS)-rDNA from *Nematodirus* was amplified using a universal primer. The forward primer was NC5: 5′-GTAGGTGAACCTGCGGAAGGATCATT-3′ and the reverse primer was NC2: 5′-TTAGTTTCTTTTCCTCCGCT-3′ [11]. PCR amplifications of the above regions were performed using 50 μL of a reaction mixture containing 22 μL of 2× Taq Mastermix (Qiagen, Hilden, Germany), 0.5 μL of each primer (50 pmol/μL), and 4 μL of template DNA; DNase/RNase-free deionized water was added to bring the volume to 50 μL. A negative control (without DNA) was included in each PCR reaction. PCR amplification conditions were as follows: initial denaturation at 94 °C for 5 min, followed by 30 cycles consisting of denaturation at 94 °C for 30 s, annealing at 45 °C for 30 s, and extension at 72 °C for 30 s. A final elongation step was conducted at 72 °C for 10 min at the end of the amplification procedure.

Based on the above PCR results, specific PCR was used to detect *N. oiratianus*. The forward primer was OLQ: 5′-GTACTCGCTGATATGGTGTC-3′, and the reverse primer was NC2: 5′-TTAGTTTCTTTTCCTCCGCT-3′ [12]. PCR amplifications of the above regions were performed using 25 μL of a reaction mixture containing 11 μL of 2×Taq Mastermix (Qiagen, Hilden, Germany), 0.25 μL of each primer (50 pmol/μL), and 1 μL of template DNA; 0.5 μL of Mg^2+^; DNase/RNase-free deionized water was added to bring the volume to 25 μL. A positive control (DNA of adult *N. longispiculata, Ostertagia* spp., *Trichostrongylus* spp., *Trichuris* spp., and *H. contortus*) and a negative control (without DNA) were included in each PCR reaction. PCR amplification conditions were as follows: initial denaturation at 94 °C for 5 min, followed by 30 cycles consisting of denaturation at 94 °C for 30 s, annealing at 60 °C for 30 s, and extension at 72 °C for 30 s. A final elongation step was conducted at 72 °C for 10 min at the end of the amplification procedure. Primers were synthesized by Shanghai Sangon Biological Engineering Technology and Services Company. All the PCR products were analyzed on 1% agarose gels and visualized using SYBR Green I. PCR products were purified using a QIAquick PCR Purification kit (Qiagen, Hilden, Germany) according to the manufacturer’s instructions and subsequently sent to TaKaRa Company for sequencing.

#### 2.3.3. Sequence Analysis

The sequences obtained were compared to those registered from NCBI under accession number KC580733.1, KC580748, KC580741, KC580742, KC580747, KC580732.1, KC580738.1 (www.ncbi.nlm.nih.gov, accessed on 28 February 2015), AF194139, AF194126, AF194140, AF194129, AF194134, (www.ncbi.nlm.nih.gov, accessed on 3 August 2000), HQ844230.1 (www.ncbi.nlm.nih.gov, accessed on 13 February 2011), JF345079 (www.ncbi.nlm.nih.gov, accessed on 31 May 2011), KR809574.1 (www.ncbi.nlm.nih.gov, accessed on 3 November 2015) by using the Basic Local Alignment Search Tool. DNA sequencing results were analyzed using the Meg Align 7.1.0 software.

### 2.4. Determination of Anthelmintic Resistance in Nematodirus

The LMIT was used to determine the anthelmintic resistance of the isolated strains [13]. The larval migration test system included the following: 18 μL of each concentration of the anthelmintic used; 40 μL of L3 larvae (about 100 larvae); 302 μL of distilled water. Ivermectin concentrations of 500, 250, 125, 62.5, 31.3, 15.6, 7.8, 3.9, 2.0, and 1.0 μg/mL were used, with four parallel wells for each concentration. As a negative control, 0.5% DMSO (Sigma, D4540, Burlington, NJ, USA) was added. These were added to a 24-well plate based on the above scheme. After incubating for 24 h at 27 °C, the liquid in each well was transferred to a transfer plate containing 0.125% agar. Motility was calculated after illumination for 48 h at 27 °C. The GraphPad Prism 5.0 software was used to analyze the data, with the logarithm of anthelmintic concentration as the *X*-axis and the rate of larval migration inhibition as the *Y*-axis. Data were fitted to the dose–effect relationship equation to obtain the dose–effect curve and EC_50_.

## 3. Results

### 3.1. Investigation of Nematodirus Infection in Sheep

**Figure 1:**
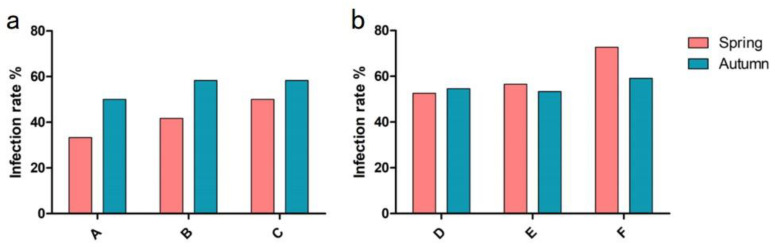
Infection rate of *Nematodirus* in Hulunbuir (**a**) and Xilingol Grasslands (**b**) before the spring and autumn deworming.

Results indicated that the *Nematodirus* infection rates of sheep in A, B, and C pastures in Hulunbuir New Barag Right Banner before the spring anthelmintic treatment were 33.3%, 41.7%, and 50.0%, respectively, with an average infection rate of 41.7%. On the other hand, the infection rates before the autumn anthelmintic treatment were 50.0%, 58.3%, and 58.3%, with an average infection rate of 55.6% (Figure 1a).

The *Nematodirus* infection rates of sheep in D, E and F pastures in the Xilinhot Blue Banner before the spring anthelmintic treatment were 52.6%, 56.5%, and 72.7%, with an average infection rate of 58.5%. On the other hand, the infection rates before the autumn anthelmintic treatment were 54.6%, 53.3%, and 59.1%, with an average infection rate of 56.3% (Figure 1b). Statistical analysis indicated no significant difference in *Nematodirus* infection rate in all surveyed pastures before the spring or autumn anthelmintic treatments.

### 3.2. Isolation and Identification of Nematodirus

#### 3.2.1. Morphology

**Figure 2:**
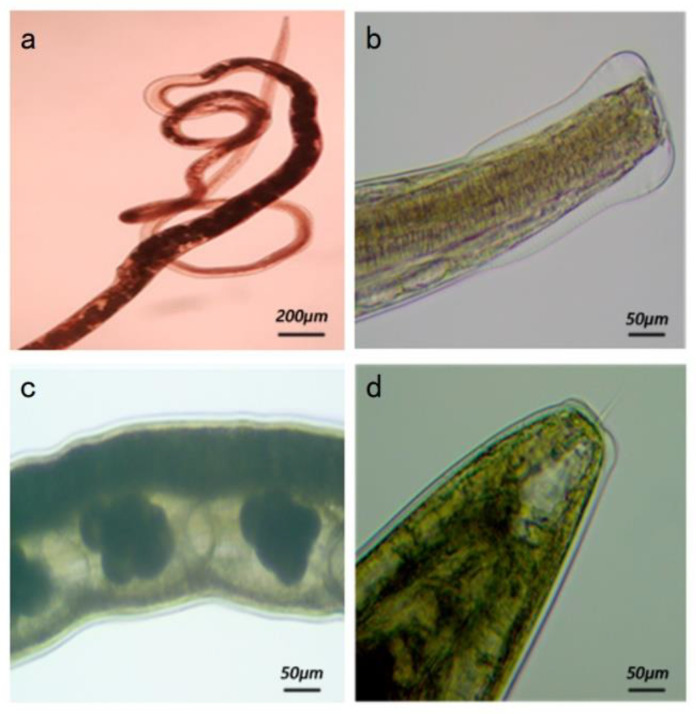
Morphology of adult *Nematodirus* spp. (**a**) Anterior segment of adult *Nematodirus* spp. (100×). (**b**) Head vesicle of adult *Nematodirus* spp. (250×). (**c**) Eggs in the uterus of a female *Nematodirus* spp. (250×). (**d**) A thin transparent thorn in the tail of a female *Nematodirus* spp. (250×).

The adult morphology of the isolated strain indicated a linear body, with its anterior end curled into a loose spiral (Figure 2a). The cephalic vesicle was observed to be wide in the front and narrow in the back, and the posterior corner of the vesicle indicated many horizontal stripes (Figure 2b). The female reproductive organs were juxtaposed with the digestive tract, and the uterus usually contained eggs (Figure 2c). The posterior end of the body indicated a truncated cone shape with a transparent spicule in the middle (Figure 2d). The male had a well-developed copulatory bursa, with two copulatory spicules of equal length. Thus, the adult morphology of the isolated strain was found to be generally consistent with the previously reported morphology of *Nematodirus* [14].

**Figure 3:**
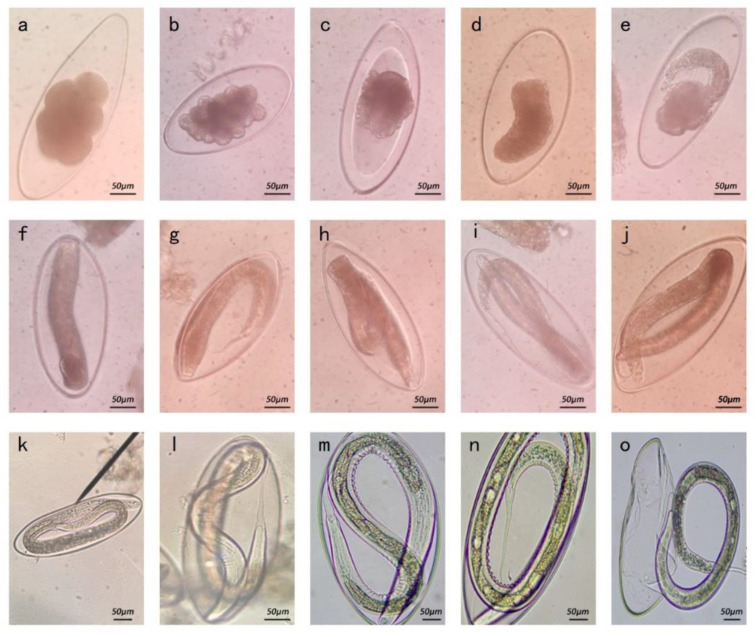
Development process of *Nematodirus* spp. (**a**) Each egg contained several egg cells, and the gap between the egg cells and the shell was large (400×). (**b**) Egg cells dividing (400×). (**c**,**d**) Egg cells begin to fuse (400×). (**e**) Embryo entering the tadpole stage (400×). (**f**,**g**) L1 larva forming (400×). (**h**–**j**) L1 larvae developing (400×). (**k**) L2 larva forming (250×). (**l**) L2 larva developing (400×). (**m**,**m**) The larva gradually developing into L3 larva (250×). (**o**) The larva hatching into mature L3 larva (100×).

Isolated strain eggs were about 102–121 × 218–312 μm in size (Table 1), which is similar to the size of *N. oiratianus* eggs [15,16]. Further observation showed that there were about 8–12 egg cells in fresh feces (day 1), and a large gap existed between the egg cell and shell. Many dividing egg cells were observed on day 2. On days 3–4, egg cells began to fuse; the embryo entered the tadpole stage on day 5. Larvae generally reached L1 on days 6–7. The larvae continued to develop into L2 larvae on about day 11. On days 12–14, they gradually developed into L3 larvae, and on day 15, some eggs hatched into mature L3 (Figure 3).

**Figure 4:**
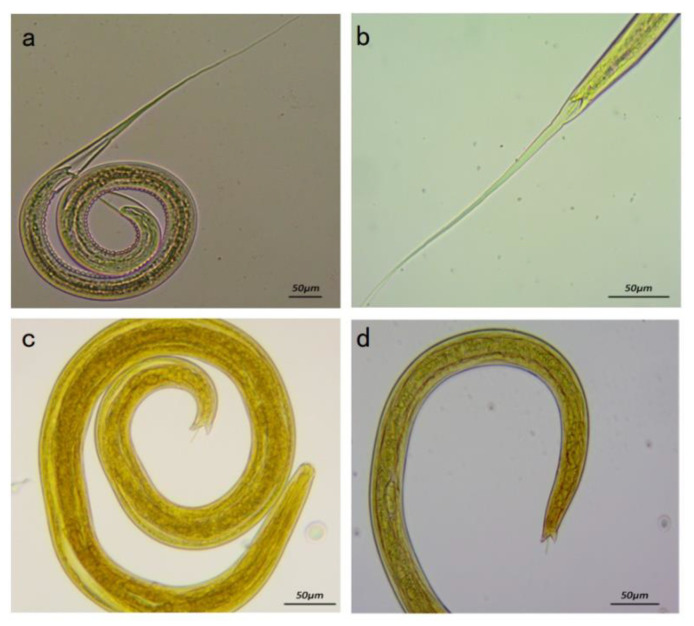
Morphology of L3 of *Nematodirus.* spp. (**a**) L3 larva (100×). (**b**) Tail of L3 larva (100×). (**c**) Exsheathed L3 larva (250×). (**d**) Tail of exsheathed L3 larva (250×).

The tails of the L3 of different *Nematodirus* species vary in their typical characteristics [17]. Microscopic observation of the morphology of third-stage larvae of the isolated strains indicated that the tail had an angular gap, and the worm had a thin spicule in the center (Figure 4). This is generally similar to the tail-end morphology of the larvae of several common species of *Nematodirus* [18]. Based on egg size and larval and adult morphology, the isolated strain was initially identified as *Nematodirus oiratianus* (*N. oiratianus*).

#### 3.2.2. Subsubsection

**Figure 5:**
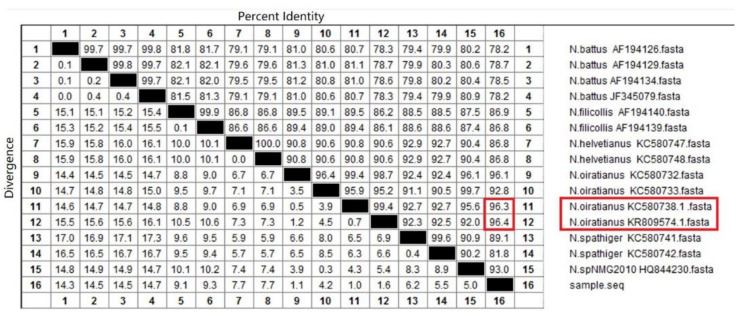
Homology alignment result based on ITS-rDNA sequences. The red box indicates that the isolated strain has high homology with *N. oiratianus*.

**Figure 6:**
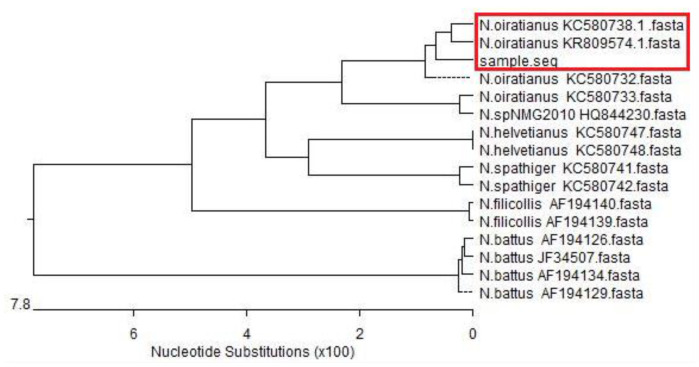
Phylogenetic tree of *Nematodirus* spp. based on ITS-rDNA sequences. The red box indicates that the isolated strain and *N. oiratianus* coalesced into one lineage.

**Figure 7:**
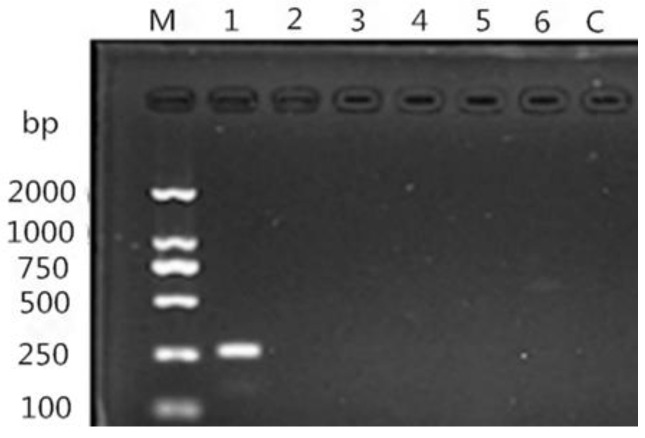
The results of specific PCR for *N. oiratianus.* M: DNA Marker LD2000; 1: *N. oiratianus*; 2: *N. longispiculata*; 3: *Ostertagia* spp.; 4: *Trichostrongylus* spp.; 5: *Trichuris* spp.; 6: *Haemonchus contortus*; C: blank control.

The ITS-rDNA gene of the isolated strain was amplified and sequenced, revealing an 800 bp sequence. Homology alignment results with *Nematodirus* ITS-rDNA sequence fragments obtained through the NCBI indicated that the highest homology was with *N. oiratianus* (KR809574. 1) at 96.4% (Figure 5). Phylogenetic tree results indicated that the ITS-rDNA sequence of the isolated strain was in the same clade as that of *N. oiratianus* (KC580738.1, KR809574.1) (Figure 6) and in a different clade from other *Nematodirus* spp. in the database. Specific PCR results also indicated that bands of about 250 bp were amplified, and non-specific bands did not exist. No obvious bands were observed in the control group (Figure 7). The results thus indicated that this specific PCR method could effectively distinguish *N. oiratianus* from *N. longispiculata, Ostertagia* spp., *Trichostrongylus* spp., *Trichuris* spp., and *H. contortus*, further confirming that the isolated strain indeed belonged to *N. oiratianus*.

### 3.3. Anthelmintic Resistance Testing (LMIT)

**Figure 8:**
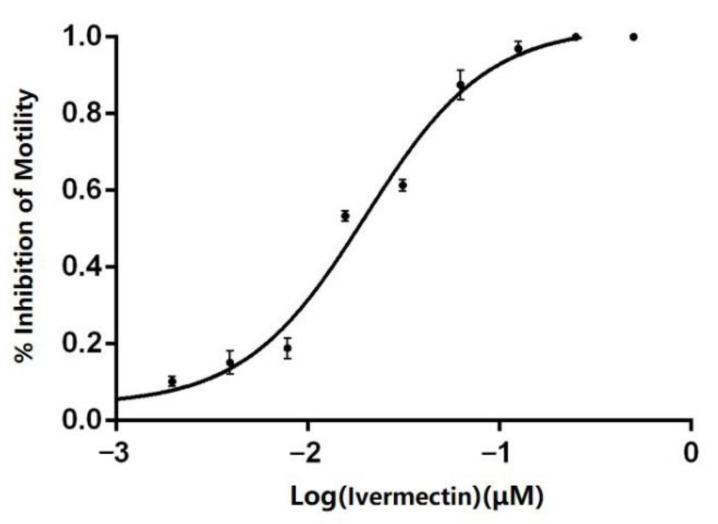
Larva migration inhibition test results.

Douch et al. used the LMIT to demonstrate that a *Nematodirus* strain could be considered ivermectin-resistant if the EC_50_ of ivermectin resistance was greater than 9.85 μg/mL for that strain [19]. Figure 8 presents the dose–effect curve of the isolated strain of *N. oiratianus*. The calculated EC_50_ is 19.98 μg/mL, indicating that the isolated strain is ivermectin-resistant.

## 4. Discussion

Nematodiriasis is found globally and has a relatively high infection rate, especially in Inner Mongolia, China. The present study investigated the prevalence of nematodiriasis in spring and autumn in major pastures of Inner Mongolia and conducted a statistical analysis of the results. The Hulunbuir and Xilingol Grasslands of Inner Mongolia were found to have serious *Nematodirus* infections, with an average infection rate of over 50%. Although the two surveyed regions employ different farming patterns, biannual anthelmintic treatment in the spring and autumn could not prevent and control nematodiriasis effectively in both semi-grazing or complete grazing pasture flocks. This demonstrates the severity of *Nematodirus* infection and anthelmintic resistance among sheep in Inner Mongolia, China.

In recent years, single nematode species from sheep with mixed gastrointestinal nematode infections have been isolated, and the morphological characteristics, host pathogenicity, and anthelmintic resistance of single nematodes have been studied. However, few studies on *Nematodirus* have been relevant. In the present study, a strain of *N. oiratianus* was successfully isolated by floating eggs in saturated saline and collecting them using a mesh. Conventional morphological classification and molecular biology were used to identify the strain as *N. oiratianus*. Observation of the egg and larval morphology of the isolated *N. oiratianus* indicated that the eggs were the largest of those in the genus *Nematodirus*; this finding is consistent with previous reports [15]. Boulenger [20] first discovered that *N. filicollis* larval development comprised three stages (L1-L2-L3), which were completed in the eggs. Later studies reported that other *Nematodirus* species also completed the three stages of larval development in eggs [16,20,21]. Similarly, the present study also confirmed that *N. oiratianus* larvae complete three stages of larval development in eggs. Furthermore, we found that *N. oiratianus* L3 larvae have angular gaps in the tail, and some worms have a thin spicule in their centers. Based on this feature, they could be distinguished from other *Nematodirus* species [17]. Wang et al. [22,23] successfully isolated *Trichostrongylus colubriformis* and *H. contortus* from mixed gastrointestinal nematode infections by identifying the unique structures of their L3 larvae. However, this method is time-consuming and requires skilled operation. Therefore, in the present study, we started with a relatively large number of *Nematodirus* eggs that were isolated and artificially infected into sheep to obtain a single-species animal infection model [24].

The body morphology, egg size, larval morphology, and other characteristics of nematodes are often used as the basis for their classification [25]. However, the morphology and size of different species of nematode eggs can be relatively similar. There is a lack of knowledge and practical experience in parasite morphology identification, and therefore, it is sometimes difficult to accurately determine the species through morphological identification alone. The ITS-rDNA gene sequence plays an important role in the identification and classification of parasites and other pathogens [26]. ITS rDNA has been reported to be used for phylogenetic analysis of *Bunostomum phlebotomum* and *B. trigonocephalum* [27]. Zhao et al. [28] identified the differentiated three species of *Nematodirus* in sheep and goats based on ITS rDNA sequences. However, Leblanc et al. [29] obtained false positive results when using universal ITS-rDNA primers for pinworm testing. Therefore, a combination of morphological observation and molecular biology can improve the accuracy of such identification. In the present study, the isolated strains were morphologically identified, and their ITS-rDNA genes were amplified and sequenced. ITS rDNA gene sequence analysis results indicated that the isolated strains had 96.4% homology with KR809574.1 but less than 90% homology with the ITS-rDNA gene sequences of other *Nematodirus* species. Phylogenetic tree analysis results also indicated that the isolated strain is in the same clade as KC580738.1 and KR809574.1 and is obviously different from other species in the *Nematodirus* genus, further confirming that the isolated strain belonged to *N. oiratianus*.

At present, resistance of sheep gastrointestinal nematodes to commonly used anthelmintics is a serious problem. It is reported that the *Nematodirus* spp. had different degrees of drug resistance to ivermectin and albendazole in sheep from 10 pastures in Inner Mongolia [6]. After treatment with ivermectin, the average *Nematodirus* spp. egg loss rate was less than 10% [4]. Appropriate methods for accurate and timely detection are essential for the prevention and control of anthelmintic resistance. However, common methods such as the fecal egg count reduction test and the larval development test are unsuitable for detection of ivermectin resistance in *Nematodirus* because of the small number of eggs laid and the larval development occurring inside the egg [30,31]. However, the LMIT only requires L3 larvae with good viability for detecting ivermectin resistance; therefore, LMIT was used to detect ivermectin resistance in the isolated *Nematodirus* strains. The results indicated that the EC50 of the naturally occurring isolated strain was 19.98 μg/mL, suggesting that it had developed severe resistance to ivermectin. The strain originated from the pasture where the epidemiological investigation was conducted, indicating that long-term use of ivermectin, albendazole, and other anthelmintics in the region had led to the development of anthelmintic-resistant *Nematodirus*. This may also account for the high infection rate in the region even after annual anthelmintic treatments.

## 5. Conclusions

The present study investigated nematodiriasis in sheep in two well-known grassland areas in China and successfully isolated a naturally occurring *Nematodirus* strain. Morphology, molecular biology, and LMIT were used to identify the isolated strain as an ivermectin-resistant strain of *N. oiratianus*. This is the only naturally occurring strain of *N. oiratianus* with anthelmintic resistance that has been isolated for the first time in many years, thus compensating for the lack of research on *N. oiratianus*. The present study provides valuable species resources and useful tools for further studies on anthelmintic resistance, thereby paving the way for a better understanding and management of *N. oiratianus* and providing basic data for the research, prevention, and control of nematodiriasis.

## Figures and Tables

**Table 1:** Statistics on egg size of *Nematodirus* spp.

Species	Longitudinal Diameter of Eggs (μm)	Transverse Diameter of Eggs (μm)	Reference
Isolated strain	261 (218–312)	111 (102–121)	Present study
*N. oiratianus*	255–272	119–153	[15]
*N. spathiger*	255–272	90–105	[16]
*N. filicollis*	140–165	70–85	[16]
*N. abnormalis*	130–220	90–119	[15]
*N. helvatianus*	160–230	85–121	[15]

## Data Availability

Not applicable.
